# Interspecies data mining to predict novel ING-protein interactions in human

**DOI:** 10.1186/1471-2164-9-426

**Published:** 2008-09-18

**Authors:** Paul MK Gordon, Mohamed A Soliman, Pinaki Bose, Quang Trinh, Christoph W Sensen, Karl Riabowol

**Affiliations:** 1 Department of Biochemistry & Molecular Biology, Faculty of Medicine, University of Calgary, Calgary, Alberta, Canada; 2 Department of Oncology, Faculty of Medicine, University of Calgary, Calgary, Alberta, Canada; 3 Department of Biochemistry, Faculty of Pharmacy, Cairo University, Cairo, Egypt

## Abstract

**Background:**

The INhibitor of Growth (ING) family of type II tumor suppressors (ING1–ING5) is involved in many cellular processes such as cell aging, apoptosis, DNA repair and tumorigenesis. To expand our understanding of the proteins with which the ING proteins interact, we designed a method that did not depend upon large-scale proteomics-based methods, since they may fail to highlight transient or relatively weak interactions. Here we test a cross-species (yeast, fly, and human) bioinformatics-based approach to identify potential human ING-interacting proteins with higher probability and accuracy than approaches based on screens in a single species.

**Results:**

We confirm the validity of this screen and show that ING1 interacts specifically with three of the three proteins tested; p38MAPK, MEKK4 and RAD50. These novel ING-interacting proteins further link ING proteins to cell stress and DNA damage signaling, providing previously unknown upstream links to DNA damage response pathways in which ING1 participates. The bioinformatics approach we describe can be used to create an interaction prediction list for any human proteins with yeast homolog(s).

**Conclusion:**

None of the validated interactions were predicted by the conventional protein-protein interaction tools we tested. Validation of our approach by traditional laboratory techniques shows that we can extract value from the voluminous weak interaction data already elucidated in yeast and fly databases. We therefore propose that the weak (low signal to noise ratio) data from large-scale interaction datasets are currently underutilized.

## Background

Protein-protein interactions play vital roles in regulating protein function and can provide valuable insight into the biological activity of proteins and biochemical pathways in which they function. The importance of protein interactions in biology has fueled intense efforts to identify such interactions and a vast repository of data has been accumulated over the years, particularly in relatively simple model organisms that are easier to manipulate genetically and biochemically. A number of bioinformatics-based approaches attempt to predict interactions using various techniques.

The budding yeast, *Saccharomyces cerevisiae*, is one of the most comprehensively studied eukaryotic organisms and a substantial amount of biochemical and genetic data has been accumulated. After the publication of the *S. cerevisiae *genome a decade ago [1], high throughput genetic and proteomic screens aimed at identifying novel genetic and protein interactions began complementing more traditional biochemical approaches [2,3]. We suspected that the voluminous data from yeast genes with human counterparts could be exploited more fully to provide better insights into human protein-protein interactions. Yeast and humans represent extreme ends of the eukaryotic evolutionary spectrum. Therefore the genes they share are often involved in fundamentally important cellular processes and represent an interesting set of genes which warrant further investigation. An example of a shared gene of particular interest to us was ING1.

The founding member of the ING family of type II tumor suppressors (ING1) was discovered using the method of subtractive hybridization aimed at identifying factors that were differentially expressed in normal mammary epithelial cells as opposed to breast cancer cell lines [4]. Ectopic over-expression of ING1 was subsequently observed to promote G1 arrest and suppression of its expression led to transformation *in vitro *and tumor formation *in vivo*. Other ING genes (i.e., ING2, ING3, ING4, and ING5) have been subsequently identified in various mammalian genomes [5]. A significant number of tumors, either (i) harbour mutations within the ING genes, (ii) have reduced expression of ING proteins, or (iii) have altered ING protein subcellular localization. A large spectrum of cancers show attenuation of ING expression (reviewed in [6,7]) and mechanistic studies have implicated the ING family in apoptosis, gene expression, senescence, hormone signaling and angiogenesis among others (reviewed in [8]). The major mechanism through which ING proteins exert their effects is through altering chromatin structure by regulating HAT and HDAC activity [9-11]. This involves binding to differentially methylated histone H3 via the ING PHD domain and also via binding to phosphatidylinositol monophosphates through the polybasic region near the PHD [12-15]. Binding is then believed to target associated HAT or HDAC complexes to chromatin regions, resulting in alteration of local histone acetylation states [10,12,14]. Other regions of the INGs have been shown to bind to PCNA, 14-3-3, [16,17] and cytoplasmic proteins such as liprin [18]. Therefore, we wished to establish a comprehensive list of ING interacting proteins that would aid in the understanding of the complex role of this family of tumor suppressors in regulating diverse cellular functions. ING genes are evolutionarily conserved and members of the ING family have been identified across the animal and plant kingdoms including the yeast *S. cerevisiae *[5].

Krogan *et al. *(2006) have described the use of tandem affinity purification (TAP) tagging followed by two different mass spectrometry methods, namely MALDI-TOF and LC-MS/MS, with the aim of assigning interacting partners to each of the yeast proteins [19]. An impressive 72% coverage of the predicted yeast proteome was made possible due to the increased sensitivity of tandem affinity purification followed by mass spectrometry. We have used this study as the initiation point to determine the range of proteins that can interact with the ING family of proteins. Here we attempt to elucidate human ING protein interactions based the recently published yeast interactome data [19], hoping to identify real interactions in the long tail of low probability noisy interactions detected.

## Results

### Pairwise alignment of YNGs (yeast ING-like proteins) and human INGs

The first member of the ING family (ING1) was discovered in humans [4] and subsequently four more ING genes have been identified (ING2-5). Homologs of ING proteins also exist throughout the animal and plant kingdoms [5]. Three yeast proteins, YNG1, YNG2 and YNG3 (Pho23), have been shown to bear considerable homology to the human ING1 protein in their C-terminal region and could functionally substitute for each other [9]. Here we generated Needleman-Wunsch pairwise alignments between individual yeast and human ING proteins. [20]. Sequences of ING1-5 (including all known ING1 isoforms) and YNG1-3 were obtained from the NCBI Genbank database . Additionally, CLUSTAL-W [21], T-COFFEE [22] and Geneious (Biomatters Ltd., NZ) were used to generate multiple sequence alignments, from which we derived the additional pairwise alignments shown in the Additional file 1. Although the alignment scores were very close to each other, given the consistency of the results obtained from the various sequence alignment tools used, the following observations can be made: (i) YNG1 shows the highest degree of sequence homology to ING1, (ii) YNG2 shows considerable homology to ING4 and ING5, and (iii) Pho23 and ING3 are similar to each other. These results agree with previous reports of phylogenetic relationships among ING proteins [5] and also with a recent report which attempts to classify ING proteins with respect to their association with either HAT or HDAC complexes [11].

### ING-interacting protein prediction

**Figure 1:**
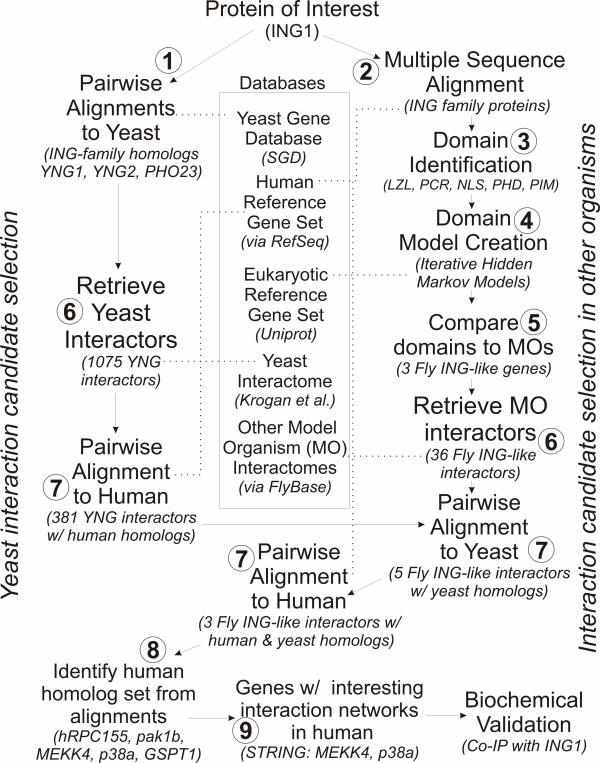
**General workflow for identifying possible protein interaction candidates for a given gene of interest (GOI), which is ING in our study.** The particular databases and genes used are given in italics.

Since members of the ING family of tumor suppressors show significant sequence conservation from yeast to humans [5], we proposed that functional interactions might also be conserved. From the available yeast interactome data, it is evident that the yeast counterparts of the ING proteins, also referred to as YNGs, interact with several other yeast proteins under normal physiological conditions [19]. We reasoned that although the majority of these interactions have very low probability scores, and hence are likely artifacts of the detection method, several of them may be transient, but nonetheless real interactions. Because of the availability of a large amount of marginal, unanalyzed yeast interaction data [19], we hypothesized there was potentially valuable untapped data to guide selection of human ING-interacting protein candidates. The yeast dataset has the advantage of being near saturation with regards to interactome coverage, so that almost all real interactions should be detected. Our confidence was bolstered by the fact that many of the previously validated ING interactions in humans were also present in the yeast interactome data. We attempted to reconcile interactomes from multiple model organisms based on two different approaches: orthology [23] and interaction network topology techniques [24]. Neither provided new insights for novel ING interacting proteins. Given the richness of available yeast data, we designed a new approach to better predict ING interactions. The bioinformatics workflow devised to filter down the massive lists of yeast interactions to a few salient candidates for biochemical validation is illustrated in Figure 1, and can be generalized to be useful for many other proteins.

### Identification of domains

**Figure 2:**
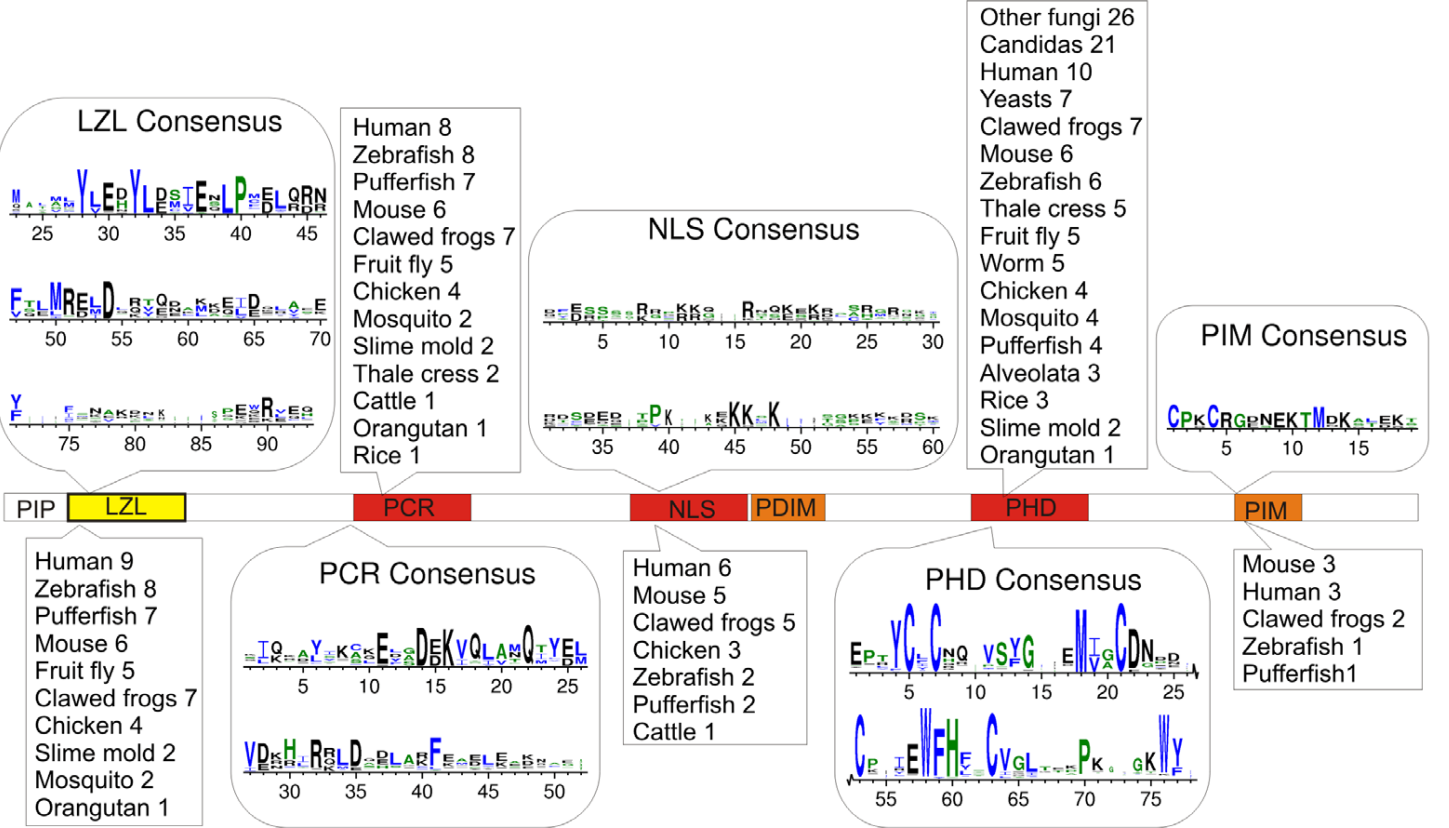
**Domain consensus and phylogenetic distribution of ING domains, based on iteratively-built Hidden Markov Models, seeded with multiple sequence alignments from **[**5.**] The height of letters represents the degree of conservation, according to the default WebLogo algorithm [67].

Since conservation of interacting partners is often a function of conservation of domain structure within [25] or across species [26,27], the next step was to identify the domain structure similarities between human and yeast ING family proteins. In order to characterize possible interaction domains of ING-like proteins quantitatively, we used an iterative consensus building processes. This process consisted of building initial Hidden Markov Models (HMMs), a position-specific amino acid substitution model of previously identified domains such as the leucine zipper-like (LZL) motif, plant homeodomain (PHD), potential chromatin regulatory (PCR) domain [now referred to as the lamin interacting domain or LID], nuclear localization signal (NLS), and peptide-interacting motif [PIM, now referred to as the poly basic region or PBR] using the multiple sequence alignments reported in [5]. These domain models were searched against the Uniprot database  (which consists of non-redundant protein datasets for all species) to identify proteins with domains closely related to the human INGs. All Uniprot proteins matching the human domain models were then added to the original model sequence to make them less species-specific and new HMMs were built based on the expanded list of sequences. This process was repeated until no new Uniprot matches were found. Because Uniprot contains data from many species, the iterative approach is a method to create domain models capturing sequence conservation amongst multiple species. The phylogenetic distribution and consensus sequences for the domains are illustrated in Figure 2, and significance thresholds are discussed later in this section.

### The use of domain-specific models

Since protein-protein interactions are primarily based on specific domains, we tried to generate domain-specific models of amino acid substitutions for the various annotated domains of the ING family proteins. This allowed us to statistically examine the validation of the domain models across species. Results obtained from this method of analysis should be more sensitive and verifiable than the generalized substitution rates used by the current ortholog detection methods based on pairwise alignments. This improved sensitivity may be due to the accounting for the specific evolution of individual protein domains and/or the greater flexibility of HMMs over simple pairwise alignments. The domain structure models generated using this approach were then used to identify possible ING-like proteins in model species for which interaction data is readily available. We investigated *D. melanogaster*, *C. elegans*, and *S. cerevisiae *interactome data as these species have the most extensively consolidated lists of protein-protein interactions.

**Figure 3:**
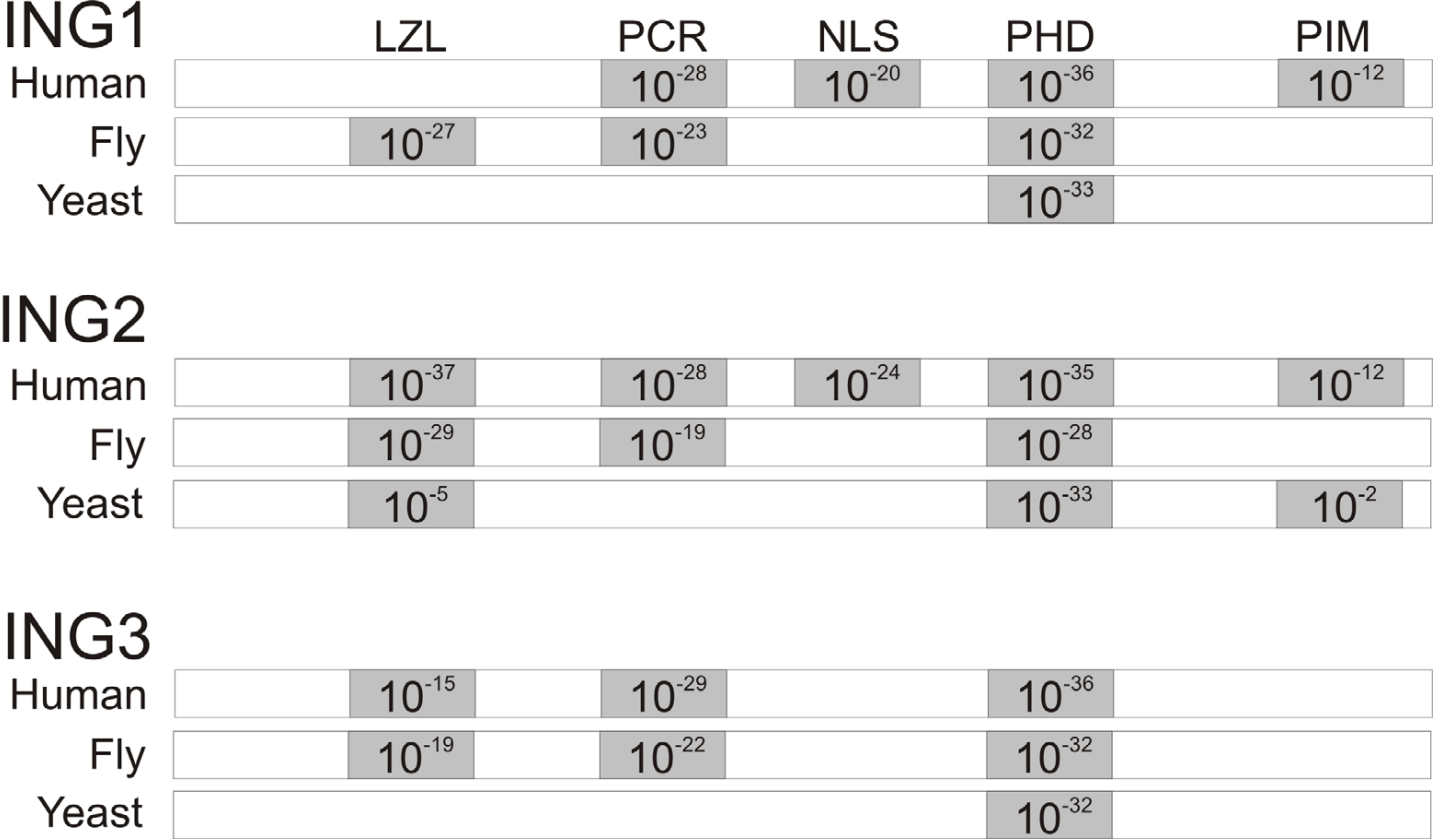
**Protein Domain scores, based on Hidden Markov Models in Figure 2 for the three species under study.** The conservation of multiple ING domains amongst the species increases the confidence in predicting conserved protein interactions among them.

We ran our generalized ING domain models against the proteomes of the above mentioned three species to verify if counterparts of human ING protein domains exist in them. The interaction databases to use depend on the conservation breadth of the gene to be investigated. Only one of the domains was found to be conserved in a single protein in *C. elegans *(PHD in Y51H1A.4, human ING1b homolog). Much better conservation of multiple ING domains was observed in *S. cerevisiae *and *D. melanogaster*. We therefore focused on identifying potential ING-interacting proteins in these two species. The domain conservation in these species is illustrated in Figure 3. As expected, the PHD domain is highly conserved across all three, with the highest overall homology with human ING2. Also noteworthy is that the fly ING1 homolog contains an LZL domain, whereas the human version does not. The PCR domain is strongly conserved in all humans and fly INGs, but is not present in yeast. It has recently been shown to interact specifically with lamin proteins and was subsequently renamed the lamin interaction domain (LID). Interestingly, the PIM domain is weakly conserved in yeast's ING2 homolog, but not in fly. The PIM was also recently renamed the polybasic region (PBR) since it harbours several basic residues and specifically binds signaling phospholipids [15]. The inclusion thresholds for each domain model were: PIM 10^-4^, PHD 10^-3^, NLS 10^-6^, PCR 10^-3^, and LZL 10^-5^. The thresholds represent the weakest e-value for any sequence used to create the domain model, i.e. the lowest score for a known positive example. The different thresholds are a consequence of the varying natures of the domain models (length, amino acid composition, phylogenetic distribution, etc.). With the exception of PIM (10^-2^), all domain matches are well above these thresholds. PIM's somewhat weak score may reflect the fact that the domain model was built using only animal sequences (Figure 2).

### Identifying human orthologs

**Figure 4:**
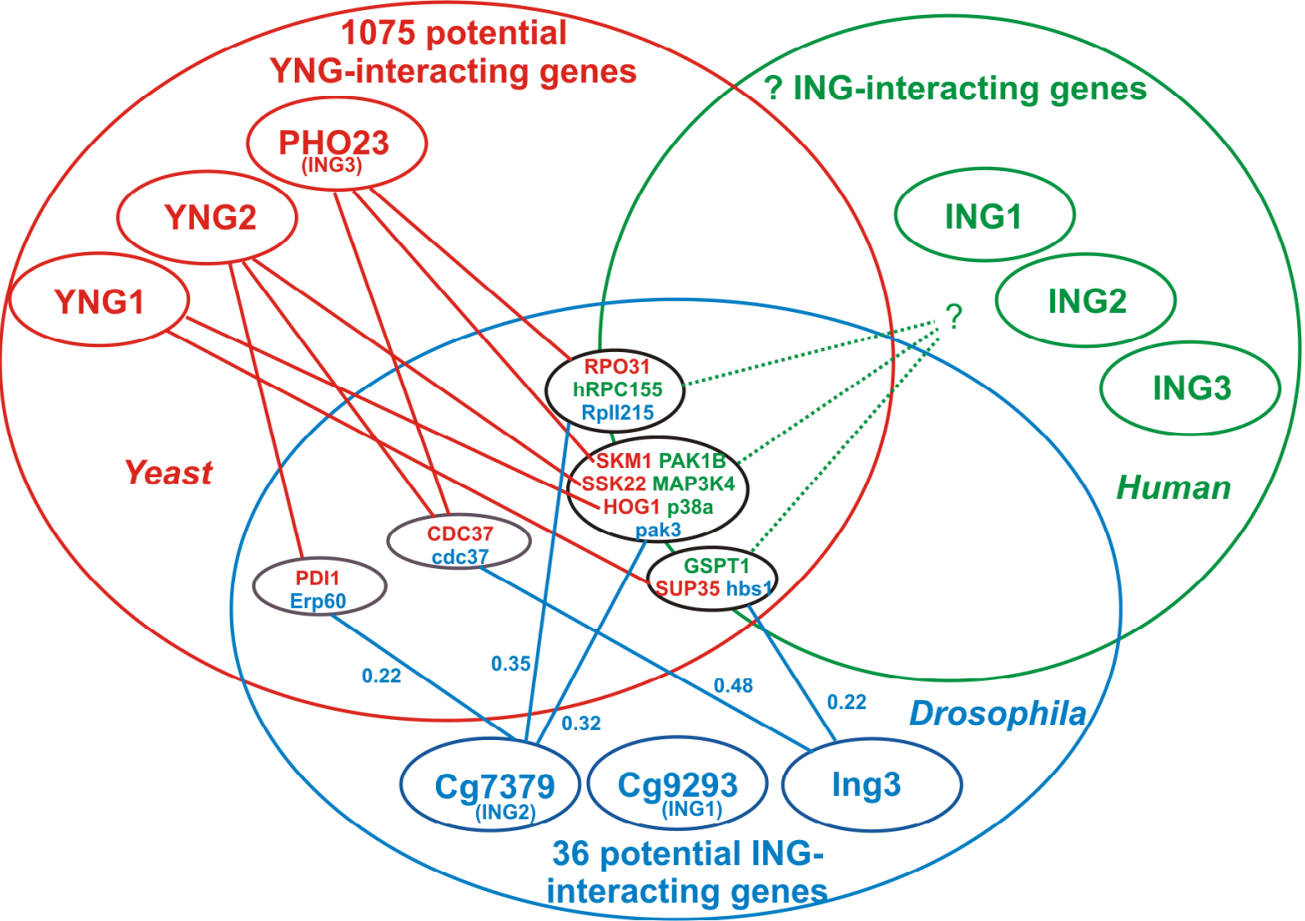
**Overlap of the ING-interactome datasets for human, fly and yeast. 5 potential ING interactions are shared between fly and yeast, and 3 of the fly interactions involve genes with good human homologs (central nodes).** These interactions (3 in fly with 5 equivalents in yeast) are candidates for biochemical validation, hence the question marks, in humans.

Using the taxonomic search tools of MAGPIE [28,29], the 1075 yeast genes were filtered to just those with human homologs. This left 381 genes that both interact with YNGs in yeast, and have human orthologs (see Additional file 2). We reasoned that the probability of the yeast interaction being conserved in humans would be higher for those proteins that show homology in another higher eukaryote because this would be evidence for the maintenance of the interaction in the Metazoan lineage. We filtered the ING-interacting proteins found in both yeast and human against the Drosophila database as the Drosophila ING showed high degree of domain conservation with the yeast and human ING proteins. Of the 36 ING-interactors identified in fly by FlyBase [30], only 5 had strong yeast homologs (e-value < 10^-35^), and only 3 of these showed a high degree of sequence conservation in humans. These 3 fly genes (having putative conserved interacting partners in yeast) have 5 potential homologs in human, namely: hRPC155, PAK1B, MAP3K4 (MEKK4), p38MAPKa, and GSPT1. The Venn diagram in Figure 4 shows the overlapping sets of potential ING-interacting proteins in fly, yeast and human. The numbers shown beside the interactions involving fly represent interaction probability [31]. All yeast interactions shown are 0.014 or 0.012 using the probability scale from the data in [19]. The homology statistics for yeast, fly and human ING-interactors that were used to construct the Venn diagram can be found in Additional file 3.

### Biochemical validation of potential human ING protein interactions

In order to select candidates for biochemical validation of human ING interactors, we compared our data with experimentally validated ING interactions in human, as listed in the STRING database [32]. Nine of the ten experimentally validated human ING interactors with yeast homologs in the 381 gene list had extremely weak interactions (p < 0.017) [19]. It therefore seems reasonable to biochemically validate any of the 5 potential human homologs, even though they had similarly low probability scores according to available yeast data. The fact that none of the 5 candidate human homologs were found in the validated list of ING interaction from the STRING database is not surprising, since the human interactome dataset is at present not nearly as saturated as that of yeast.

To restrict the list of 5 candidates further, we considered the biological relevance of the potential interactions to the known functions of INGs. Accordingly, the choice was amongst PAK1b, MAP3K4 (MEKK4) and p38MAPKa in descending order of homology among the 3 species (e-values 10^-98^, 10^-45^, and 10^-26^, respectively). We also selected another gene (RAD50) that does not fulfill all of the requirements of our method, but which is predicted by STRING. RAD50 has a weak yeast interaction score but an extremely strong homolog (10^-156^) in humans. We wanted to test if in such cases the yeast data could be used alone in successfully predicting human ING interactors.

**Figure 5:**
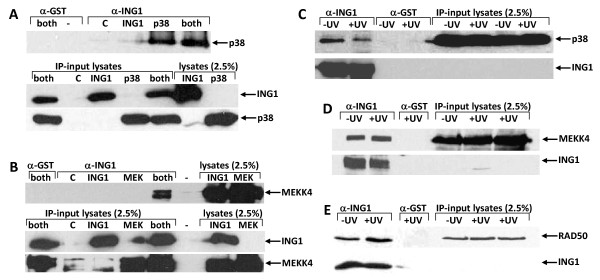
**Biochemical validation of potential human ING protein interactions. A) HEK293 cells were transfected with the equal amounts of the indicated constructs and cell lysates were immunoprecipitated using anti-ING1 and then immunoblotted with anti-p38MAPK.** The blot was reprobed with anti-ING1 to confirm equal IP efficiency. B) Same as in panel A but immunoblotting with anti-MEKK4. C) Endogenous interaction between ING1 and p38 MAPK under non-UV and UV conditions. HEK293 cell lysates were immunoprecipitated using anti-ING and then immunoblotted with anti-p38MAPK. Interestingly, the interaction seems to be stronger under normal rather than stress conditions. The input shows equal amounts of cell lysates have been used. ING1 levels after reblotting of the same membrane with anti-ING1. ING1 levels were equal under both normal and stress conditions. D) Same as in C but the immunoblotting was done with anti-MEKK4 to detect ING1-MEKK4 interaction at the endogenous interaction. E) Same as in C but the immunoblotting was done with anti-RAD50 to detect ING1-RAD50 interaction at the endogenous level.

Based on scientific relevance of the interactions (see Discussion), we chose to biochemically validate three putative interactions (MEKK4, p38MAPK and RAD50) using co-immunoprecipitation (Co-IP) followed by western blot analysis. As shown in Figure 5A, IP-western analyses indicated that endogenous RAD50 specifically interacted with endogenous ING1. Since the other two proteins that we chose to investigate, MEKK4 and p38MAPK are closely linked in a stress pathway [33], we chose to confirm these interactions under both normal and stress conditions approaches, namely UV. Figure 5B shows that overexpressed p38MAPK and overexpressed MEKK4 showed strong signals in ING1 immunoprecipitates, but not in the negative control glutathione-S-transferase (GST) immunoprecipitates. Figure 5C shows that ING1 immunocomplexes from untransfected cells, but not GST immunocomplexes, contain both p38MAPK and MEKK4, confirming that this interaction occurs between endogenous proteins. Unlike the case for ING1-PCNA interaction that is increased by UV-induced stress [16], treatment of cells with a UV dose sufficient to induce a stress response did not markedly alter the degree of kinase-ING1 interaction. Input lanes also show that robust signals were obtained for both the p38MAPK and MEKK4 proteins in control western blots of lysates used, under conditions where little, if any, signal was seen for ING1. This indicates that high levels of the kinases are expressed in our experimental cell system, compared to ING1 protein. Relatively high expression levels of the kinases compared to ING1 likely explains why reciprocal IP-western assays did not clearly demonstrate a detectable interaction (data not shown), since only a small portion of the kinases would be expected to interact with ING1 protein based upon their apparent relative stoichiometry.

### Comparison to existing datasets and methods

To evaluate the combined contribution of Krogan *et al. *marginal data and our prediction technique to the study of protein-protein interactions, we compared our results to those obtained from biochemical surveys, and other prediction algorithms [34]. Through an evaluation of the completeness of current yeast and human protein-protein interaction networks, we propose that making raw unfiltered results available to all researchers could help distinguish between real and spurious interactions. Table 1 summarizes the few ING interactions we could extract from the available datasets surveyed by Hart *et al. *[34], covering most of the commonly used techniques, from yeast two-hybrid to tandem affinity purification followed by mass spectrometry. Unfiltered experimental data is only available in very few of the datasets listed in Table 1, therefore we observed how many of the ING interactions in other datasets also occurred in the unfiltered Krogan dataset, and how well they matched our technique's criteria. It is clear from the data that YNG/ING is poorly represented in several datasets, and that different methods produced different biases in which ING is detected. This supports the notion by Hart *et al. *that even the well-studied yeast interactome is only about 50% elucidated by existing filtered datasets.

**Table 1:** Comparison of datasets used by Hart *et al.*[34] for ING protein interaction predictions.

	**Reference**	**Total number of Interact-ions**	**ING1/YNG1 interactions**	**ING2/YNG2 interactions**	**ING3/PHO23 interactions**	**Interactions matching our predictions**
**Yeast**	Krogan [19]*	393,878	237	772	349	5
	Krogan [19]	14,317	5	24	15	0
	Jansen [36]#	49,640	0	0	0	0
	Ho [68]	8,118	0	0	0	0
	Gavin [69]	589~	0	0	3	0
	Ito [70]	4,549	2	1	2	0
	Uetz [71]	957	0	0	0	0
	Gavin [72]	491~	2	2	8	0
	von Mering [32]^	N/A	11	19	17	0
**Humans**	Rual [73]	6,726	0	0	0	0
	Stelzl [74]	5,749	0	0	0	0
	Rhodes [75]	39,816	11	0	0	0
	Lehner [76]	71,806	3	0	0	0
	von Mering [32]^	N/A	22	3	18	0

*raw dataset

~proteins purified

#using L_PIT _> 300

^medium confidence experimental reports

Based on our success in identifying valid ING interacting proteins from the unfiltered dataset in Krogan *et al.*, we strongly agree with Hart *et al. *that the research community would be much better served by the release of raw interaction datasets in general for comparison and consolidation. Additionally, some original datasets, such as those from Ito *el al.*, were only available via the Internet Archive  as the original web links referenced in the papers no longer exist.

Other bioinformatics-based approaches have been used to predict interactions between proteins (for a review, see [35]). Sequence, domain, and motif structure based approaches form the basis of Bayesian network models [36]. Examining co-evolution of interacting proteins by comparing phylogenetic trees [37], correlating mutations [38], or gene fusion [39] also rely on sequence based approaches. Protein domain interface-based approaches [40] also exist. Other approaches such as gene expression, gene ontology annotations, and transcriptional regulation, can also be used to predict whether or not a group of proteins are members of the same complex. Our attempts to use conventional protein-protein interaction prediction tools [41-43] on ING and YNG proteins did not yield results beyond those described in the various public interaction repositories (as listed in [44]) or predicted by literature text mining. An exhaustive comparison of our technique to others is beyond the scope of this paper, but Table 2 summarizes the results of searching for ING1/2/3 interactions employing various techniques.

**Table 2:** Comparison of ING results for existing protein interaction prediction tools.

**Prediction Tool**	**Total number of Interactions**	**ING1/2/3 interactions**	**Interactions not in existing STRING dataset**	**Overlap with predictions from this work**
I2D [78]	200,599	33	2	0
YeastNet [77]#	102,803	100	57	0
OPHID [41]	47,221	10	2	0
POINT [43]	45,378	49	26	1^
Ulysses [42]	26,797	22	15	1*
Predict [79]	20,088	5	5	1*

# A yeast database

^The concurring interactor, PDI1, was excluded from the 5 core predictions of this work due to weakness of the human homolog (see Figure 4)

* The concurring interactor, CDC37, was excluded from the 5 core predictions of this work due to weakness of the human homolog (see Figure 4)

It must be noted that our core predictions (hRPC155, PAK1B, MAP4K3, p38MAPK and GSPT1) do not overlap with other ortholog-based techniques [43], which would be the most natural comparison to make. Interestingly, our two marginal predictions, PDI1 and CDC37, agree with some methods in Table 2. The fact that none of the core predictions overlaps, but marginal ones do highlights the fact that different techniques were used to define orthologs. All of the methods in Table 2 used either InParanoid [45] or Homologene [46] to define interspecies gene mappings. The former maps only YNG1 and Pho23 to the human counterparts we have identified, while the latter maps all three yeast ING equivalents to ING3.

The uniqueness of our core predictions suggests that the technique we have developed provides added value over a straightforward multi-species prediction tools. Given an unfiltered dataset, it is possible that some of the techniques used in Table 2 that employ existing biochemical data would also predict some or all of our five candidate interactions. However, we are unaware of any follow-up studies by the authors of those tools using a raw dataset. It is not unreasonable to assume that the level of false positive predictions from these tools would increase substantially without some changes to their algorithms, which were built for "clean" input datasets. In contrast, we err on the side of false negatives by using strict 1) multi-species criteria and 2) gene-family specific domain models to cast a highly restrictive "lens" on Krogan *et al's *massive unfiltered dataset. This explains why our methodology is complementary to the existing techniques. We do not suggest that our technique will find all true positives, because interactions are not always shared between multiple species, and not all interactions have been elucidated. Rather, our technique provides guidance for researchers working on proteins whose interactions are not successfully predicted using existing techniques (such as the ING family presented here).

## Discussion

In this study we have shown that a high degree of conservation of the ING proteins exists between human and yeast based on their interactions with analogous proteins across these species. This is consistent with previous reports showing conservation of ING protein sequence, particularly in specific domains [5], and conservation of function in regulating chromatin structure through associations with HAT and HDAC complexes (reviewed in [8,47]). In addition to the specific interactions that we have confirmed experimentally, our work highlights the fact that many additional potential and novel interactions may occur between analogous proteins in these two organisms. Over 1,000 proteins were reported to interact with YNGs [19] and of these proteins, we found that 381 had identified homologs in human cells. Based on our MAGPIE analysis and initial examination of three of the proposed interactions, many of the set of 381 proteins are likely to also interact with human INGs. The *in silico *approach we have designed allowed us to predict new protein-protein interactions for the human INGs with a high degree of success and confirmed many previously elucidated interactions such as those with p21, Karyopherin, HAT/HDAC proteins and histone H3.

Our findings suggest that ING family proteins are involved in a more diverse array of biological processes than are presently suspected from the current literature and some of the interactions suggest possible additional mechanisms that might underlie their tumor suppressor capabilities. The three new interactions we have elucidated and biochemically confirmed here, RAD50, p38MAPK and MEKK4, further link ING1 to DNA damage/stress response pathways [8,48]. ING interaction with RAD50, an important component of the MRE11-RAD50-NBS1 complex, gives credence to previous reports linking ING proteins to DNA damage signaling and repair pathways via PCNA and GADD45 [16,49,50].

**Figure 6:**
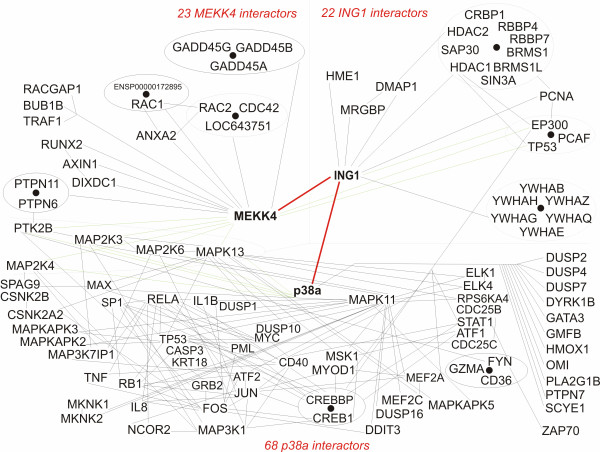
**Merger of the interaction maps for the three human proteins ING1, MEKK, and p38a (p38MAPK) based on empirical data retrieved from the STRING database.** Lines connecting p38a and its interactors have been excluded for clarity, except where the interactor is directly shared with MEKK4 as part of a well-defined stress response pathway. MEKK4 and ING1 have relative few confirmed interactions, but the interactions predicted and confirmed in our study (shown as thick lines) are the first to directly tie ING1 to multiple key proteins in this stress response. Examining such interaction overlap graphs can help, in deciding which interaction predictions to validate biochemically, based on biological tenability and salience.

In an attempt to understand the connection between the ING, MEKK4, and p38MAPK protein interaction networks, we generated a merged interaction graph (Figure 6). Several reports have indicated that different forms of stress, such as UV, chemotherapeutic agents and hypoxia affect the function of the ING proteins [15,16,51-55]. The mammalian JNK/p38MAP kinase kinase kinase (MEKK4) and the yeast (*S. cerevisiae*) Ssk2p protein are homologous, with MEKK4 being able to replace all of the known functions of Ssk2p in yeast. The stress-activated mitogen-activated protein kinase (SAPK) pathways are integral components of diverse stress signaling pathways such as UV, hypoxia, heat, osmotic shock, pH, oxidative damage, cytokines, pheromones and others [33,56]. The fact that ING1 can interact with both MEKK4 and p38MAPK is not surprising given the facts that all three proteins are evolutionarily conserved, bear common links to several different signaling pathways, both ING1 and MEKK4 bind to GADD45 [49,57] and both MEKK4 and p38MAPK are in a well defined stress response pathway [33]. This observation is also consistent with reports that ING proteins affect transcription factor activity [58,59] since the MEKK4/p38MAPK stress activated kinase cascade culminates in the regulation of various transcription factors, some of which are outlined in Figure 6. Analysis of the effects of altering ING1 activity on MEKK4/p38MAPK signaling under different conditions of extracellular and intracellular stress should serve to better clarify the roles that physical interaction of ING1 with these proteins plays in the mammalian stress response cascades. Since several other ING-interacting partners showed similar degrees of interaction, it is tempting to speculate that further examination of the additional candidate ING-interacting pathways we have identified in multiple model organisms, and particularly in yeast, will shed further light on the function of the ING family of chromatin regulators.

The overall philosophy of the prediction procedure outlined in Figure 1 is to examine the large number of interactions detected in yeast for a given protein, regardless of their probability scores, and then to reduce the list to a few candidates. This reduction is accomplished by successively keeping only the overlap of: 1) domain occurrence, and 2) interaction pairs, in at least three species. This approach stands in contrast to current automated prediction methods based on just one or two species, which often use only relatively high-scoring interaction data to prevent too many false positive predictions. The success of our approach, with the test case of ING1, suggests that the large quantity of low-scoring interaction data available in yeast is currently underutilized.

Our approach is semi-automatable (see Methods), but the researcher must: 1) assist in creating the multiple sequence alignments of domains, and 2) select biologically tenable interactions from the final shortlist generated. The procedure focuses on one gene (or set of closely related genes) at a time, and is not specific to ING proteins, but rather can be applied to any human gene with equivalents in yeast. We expect researchers who concentrate on a specific gene can beneficially use this focused approach to interaction prediction when generalized, large-scale prediction services do not yield good results.

## Conclusion

We have developed a method using comparisons in different organisms in which homologs exist, to predict with a high degree of certainty what particular protein interactions found in unfiltered data may occur *in vivo *and contribute to the activities of, in this case, the ING proteins. This cross-species (yeast, fly, and human) bioinformatics-based approach was used to identify potential human ING1 interacting proteins with higher probability and accuracy than approaches based on screens in a single species. We confirm the validity of this screen and show that ING1 interacts specifically with three proteins tested: p38MAPK, MEKK4, and RAD50. These novel ING-interacting proteins further link ING proteins to cell stress and the DNA damage signaling, providing previously unknown upstream links to DNA damage response pathways in which ING1 participates.

## Methods

### Computational approach

The process of creating a list of protein interaction predictions consists of 8 broad steps (please refer to Figure 1 for the precise data flow between steps). We assume that the researcher already has a human gene, or closely related set of human genes of interest (GOI) in mind for analysis, which in our case was ING. The first step was to determine if anything resembling ING exists in yeast. This was done using [60] against the full set *of S. cerevisiae *genes downloaded from the yeast genome database (SGD) [61]. Given good pairwise matches, we determined that it was worthwhile to proceed with step 2: the construction of domain models to provide a quantification of their conservation among species. In step 2, a multiple sequence alignment (MSA) was performed using CLUSTALW [21], and adjusted by hand as required. In step 3, the HMMER software  was used to build and calibrate Hidden Markov Models (HMMs) from multiple, distinct conserved regions, i.e. potential domains, of the MSA. In step 4, these HMMs were used to search for proteins in other species with the same domains, using a DeCypher hardware-based HMM search (ActiveMotif Inc., Carlsbad, CA), although HMMER software could also be used. The database searched against was Uniprot [62], which provides a non-redundant set of know eukaryotic genes. Each HMM's search results was reviewed by hand, and portions of database sequences deemed matching (primarily those with e-value < 10^-5^, and few large gaps) were incorporated into the HMM. This searching and extra sequence incorporation was done iteratively (since new sequences in the HMM affect e-value results) until no new matches were found in Uniprot. The end result of step 4 was that for each domain we had an HMM representing the domain's very particular evolution across eukaryotic species. Step 5 was to compare the HMMs against the complete protein sets from model organisms with large amounts of interaction data, namely, worm [63], fly [30] and yeast (SGD). This was once again done using the DeCypher HMM search, and identified model organism genes with the same domains as the ING proteins. Given HMM matches in yeast and at least one more model organism (fly, in our case), we proceeded in step 6: to extract its interacting proteins. The source of the interaction data was either 1) yeast data base [19] or 2) the other model organism database (FlyBase) [30]. Step 7 was to reduce the list of ING-interacting proteins in the model organisms to just those satisfying two conditions: 1) strong pairwise ING-iteracting proteins homology between human, yeast and fly, and 2) having interaction data in both yeast and fly. These filters reduced the list dramatically. Finally, in step 8, we viewed the human homologs of each ING-interacting proteins in the STRING database [64] and assessed the biological relevance of the potential interactions in humans.

We have focused on the use of thorough methods in our approach to maximize the sensitivity of our results. It would be possible to substitute certain methods, such as BLAST [65] for Smith-Waterman, or InterPro models searches [66] for HMM building in these steps, with the caveat of reduced predictive value of the results.

### Cell culture

HEK293 (ATCC CRL-1573) cells were maintained in Dulbecco's Modified Eagle's medium (DMEM; Gibco-RBL) supplemented with 10% fetal bovine serum and 100 units/ml of penicillin and 100 mg/ml of streptomycin (Gibco-BRL) in an incubator with 5% CO_2 _at 37°C. Cells were seeded in 10 cm or 15 cm dishes 24 hrs prior to transfection.

### Constructs

The pCI-ING1b plasmid has been described in [16], and the pTP11 (Rad50 with C-terminal *his *tag), MEKK4 and p38MAPK constructs were kind gifts from Drs. Tanya Paull (Rad50), Steve Pellech (p38MAPK), Richard Vaillancourt (MEKK4) and James Woodgett (p38MAPK).

### *In vitro *transfection and UV-irradiation

HEK293 cells were transiently transfected with the plasmids mentioned above at 60–70% confluence using a standard calcium phosphate protocol. Media was removed after 24 hrs, cells were washed with PBS and either exposed to 40 J/m^2 ^of UV radiation or left untreated. Fresh media was added and cells were incubated for 2 hrs before they were harvested.

### Co-immunoprecipitation and western blotting

Transfected and untransfected HEK293 cells were harvested and lysed under non-denaturing conditions in ice-cold RIPA buffer containing protease inhibitors (Complete Mini, EDTA-free protease inhibitor cocktail tablets from Roche Diagnostics). Cell lysates were sonicated on ice and centrifuged at 14,000 × *g *at 4°C for 15 min. The supernatants thus obtained were precleared by incubation with 20 μl 1:1 slurry of protein G-Sepharose (Amersham) for 30 min at 4°C and then incubated with 5 μg of specific antibody and 40 μl of protein G-Sepharose (1:1 slurry) or an equivalent amount of mouse anti-ING1 preconjugated with 40 μl of protein G-Sepharose (1:1 slurry) at 4°C for 3 hrs on a roller system. The immunocomplexes recovered on beads were washed two times for 5 min with 1 ml of RIPA buffer before the addition of Laemmli sample buffer. Proteins were resolved by sodium dodecyl sulfate-PAGE and transferred to nitrocellulose membranes (Hybond; Amersham). Immunoblotting was performed with a cocktail of four mouse anti-ING1 monoclonal antibodies or with rabbit anti-RAD50 polyclonal (Abcam), anti-p38MAPK (Zymed) or anti-MEKK4 (a gift from Dr. Richard Vaillancourt). Immunoreactive bands were visualized using an enhanced chemiluminescence reagent (Amersham Biosciences).

## Authors' contributions

PG devised the interspecies mining algorithm. PG and MS wrote the initial version of the manuscript. MS and PB reviewed the predictions and performed the lab experiments. QT assisted in the pairwise and multiple sequence alignments. KR conceived of the study and CS and KR provided guidance on the biological relevance of the method and manuscript revisions. All authors read and approved the final manuscript.

## Supplementary Material

Additional file 1 Pairwise similarity of ING family proteins in yeast and human. Using various alignment algorithms, we found that YNG1 is the ortholog of human ING1/2, YNG2 is the closest homolog to human ING4/5, and PHO23 (YNG3) is similar to human ING3. Click here for file

Additional file 2 Potential yeast ING-interacting proteins with human homologs. Using the taxonomic tool in MAGPIE, we filtered the list of 1075 yeast ING-interacting proteins to only those having human homologs with e-value < 10^-35^, yielding 381 potential conserved interactions in human. Click here for file

Additional file 3 Evidence for potential ING-like proteins and their interactors in worm, fly, human and yeast. In order to increase the confidence in our predictions, we filtered the human-yeast common ING interactors to only those interactions conserved in fly (worm had poor homologs). We found 36 fly ING-interacting proteins with either yeast or human homologs, and only 5 showed conservation amongst the three species. Click here for file
