# Supplementary material for: Interspecies data mining to predict novel ING-protein interactions in human
Source: BMC Genomics. 2008 Sep 18;9:426. doi: 10.1186/1471-2164-9-426 (PMC2565686; doi:10.1186/1471-2164-9-426)
Supplement: Additional file 1 — Pairwise similarity of ING family proteins in yeast and human. Using various alignment algorithms, we found that YNG1 is the ortholog of human ING1/2, YNG2 is the closest homolog to human ING4/5, and PHO23 (YNG3) is similar to human ING3. [file 1471-2164-9-426-S1.ppt]

## Slide 1
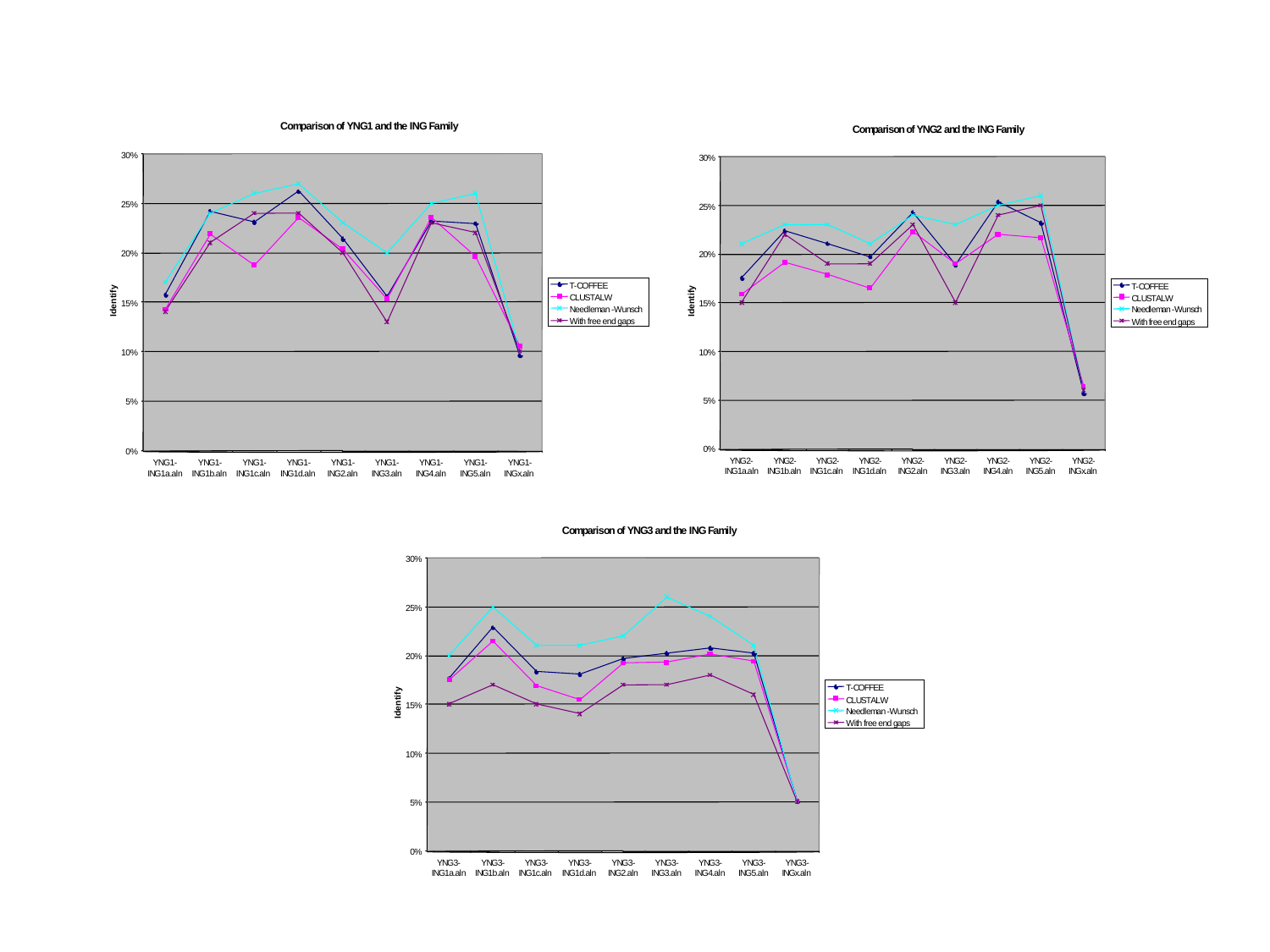

C
o
m
p
a
r
i
s
o
n
o
f
Y
N
G
1
a
n
d
t
h
e
I
N
G
F
a
m
i
l
y
3
0
%
2
5
%
2
0
%
T
-
C
O
F
F
E
E
Identify
1
5
%
1
0
%
5
%
0
%
Y
N
G
1
-
Y
N
G
1
-
Y
N
G
1
-
Y
N
G
1
-
Y
N
G
1
-
Y
N
G
1
-
Y
N
G
1
-
Y
N
G
1
-
Y
N
G
1
-
I
N
G
1
a
.
a
l
n
I
N
G
1
b
.
a
l
n
I
N
G
1
c
.
a
l
n
I
N
G
1
d
.
a
l
n
I
N
G
2
.
a
l
n
I
N
G
3
.
a
l
n
I
N
G
4
.
a
l
n
I
N
G
5
.
a
l
n
I
N
G
x
.
a
l
n
C
o
m
p
a
r
i
s
o
n
o
f
Y
N
G
2
a
n
d
t
h
e
I
N
G
F
a
m
i
l
y
3
0
%
2
5
%
2
0
%
T
-
C
O
F
F
E
E
Identify
1
5
%
1
0
%
5
%
0
%
Y
N
G
2
-
Y
N
G
2
-
Y
N
G
2
-
Y
N
G
2
-
Y
N
G
2
-
Y
N
G
2
-
Y
N
G
2
-
Y
N
G
2
-
Y
N
G
2
-
I
N
G
1
a
.
a
l
n
I
N
G
1
b
.
a
l
n
I
N
G
1
c
.
a
l
n
I
N
G
1
d
.
a
l
n
I
N
G
2
.
a
l
n
I
N
G
3
.
a
l
n
I
N
G
4
.
a
l
n
I
N
G
5
.
a
l
n
I
N
G
x
.
a
l
n
C
L
U
S
T
A
L
W
C
L
U
S
T
A
L
W
N
e
e
d
l
e
m
a
n
-
W
u
n
s
c
h
N
e
e
d
l
e
m
a
n
-
W
u
n
s
c
h
W
i
t
h
f
r
e
e
e
n
d
g
a
p
s
W
i
t
h
f
r
e
e
e
n
d
g
a
p
s
C
o
m
p
a
r
i
s
o
n
o
f
Y
N
G
3
a
n
d
t
h
e
I
N
G
F
a
m
i
l
y
3
0
%
2
5
%
2
0
%
T
-
C
O
F
F
E
E
Identify
1
5
%
1
0
%
5
%
0
%
Y
N
G
3
-
Y
N
G
3
-
Y
N
G
3
-
Y
N
G
3
-
Y
N
G
3
-
Y
N
G
3
-
Y
N
G
3
-
Y
N
G
3
-
Y
N
G
3
-
I
N
G
1
a
.
a
l
n
I
N
G
1
b
.
a
l
n
I
N
G
1
c
.
a
l
n
I
N
G
1
d
.
a
l
n
I
N
G
2
.
a
l
n
I
N
G
3
.
a
l
n
I
N
G
4
.
a
l
n
I
N
G
5
.
a
l
n
I
N
G
x
.
a
l
n
C
L
U
S
T
A
L
W
N
e
e
d
l
e
m
a
n
-
W
u
n
s
c
h
W
i
t
h
f
r
e
e
e
n
d
g
a
p
s
