# Supplementary figures and images for: Interspecies data mining to predict novel ING-protein interactions in human
Source: BMC Genomics. 2008 Sep 18;9:426. doi: 10.1186/1471-2164-9-426 (PMC2565686; doi:10.1186/1471-2164-9-426)

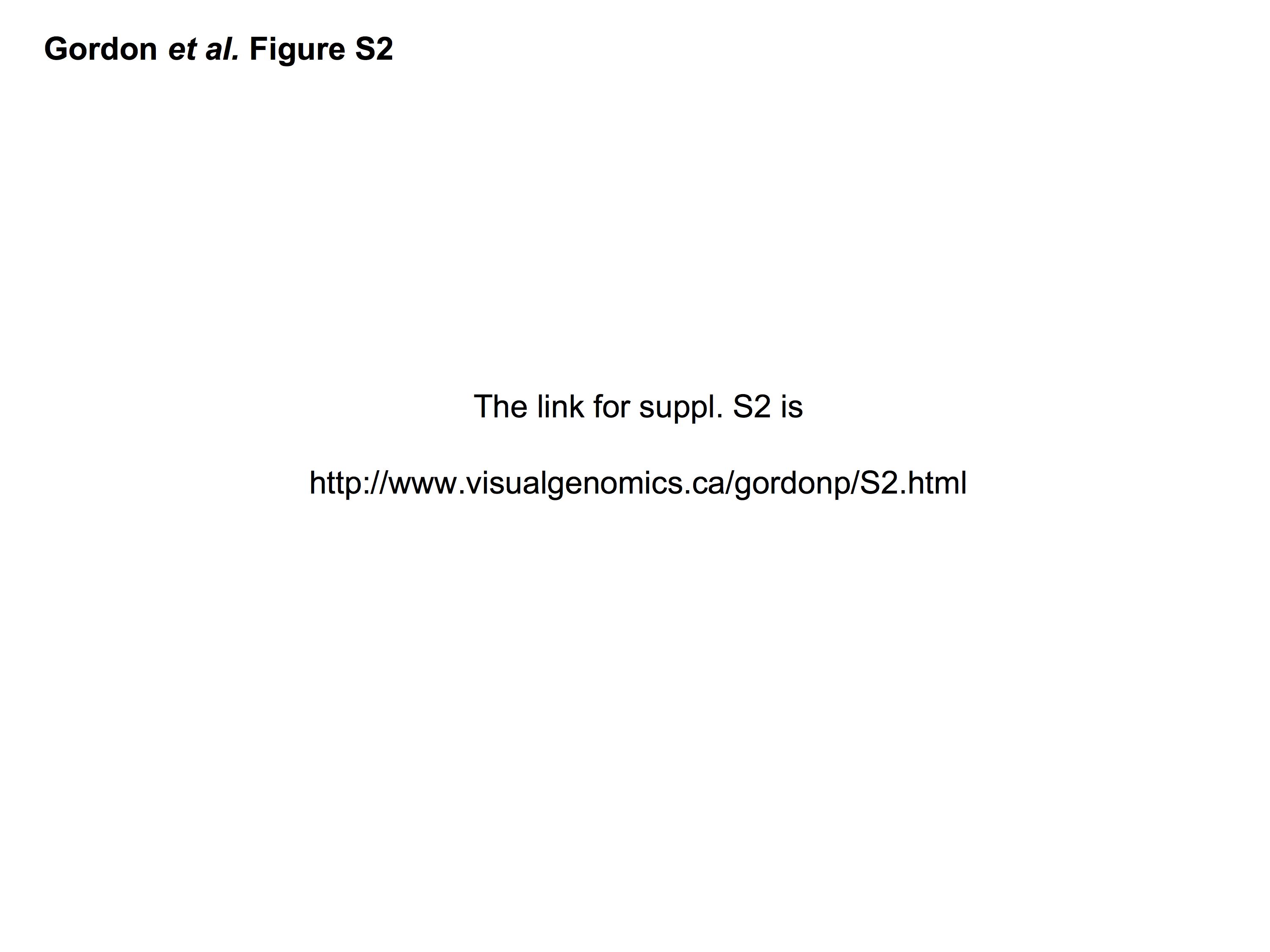

Supplement: Additional file 2 — Potential yeast ING-interacting proteins with human homologs. Using the taxonomic tool in MAGPIE, we filtered the list of 1075 yeast ING-interacting proteins to only those having human homologs with e-value < 10-35, yielding 381 potential conserved interactions in human. [file 1471-2164-9-426-S2.jpeg]
