# Supplementary material for: Interspecies data mining to predict novel ING-protein interactions in human
Source: BMC Genomics. 2008 Sep 18;9:426. doi: 10.1186/1471-2164-9-426 (PMC2565686; doi:10.1186/1471-2164-9-426)
Supplement: Additional file 3 — Evidence for potential ING-like proteins and their interactors in worm, fly, human and yeast. In order to increase the confidence in our predictions, we filtered the human-yeast common ING interactors to only those interactions conserved in fly (worm had poor homologs). We found 36 fly ING-interacting proteins with either yeast or human homologs, and only 5 showed conservation amongst the three species. [file 1471-2164-9-426-S3.doc]

ING family Domain Hits in Drosophila (fly ING homologs)

**LZL** (4 proteins in Drosophila with LZL-like domains)

| RANK | SCORE | LOCUS | E_VALUE |
| --- | --- | --- | --- |
| 1 | 109.91 | CG7379-PA | 1.6e-29 *[Human ING2 homolog (source: InParanoid)]* |
| 2 | 101.75 | CG9293-PB | 4.5e-27 *[Human ING1b homolog (source: flybase)]* |
| 3 | 101.75 | CG9293-PA | 4.5e-27 |
| 4 | 74.40 | Ing3-PA | 7.8e-19 *[Human ING3 homolog (source: InParanoid)]* |

**PCR** (4 proteins in drosophila with PCR-like domains)

| *RANK* | *SCORE* | *LOCUS* | *E_VALUE* |
| --- | --- | --- | --- |
| 1 | 87.63 | CG9293-PB | 8.1e-023 |
| 2 | 87.63 | CG9293-PA | 8.1e-023 |
| 3 | 84.07 | Ing3-PA | 9.5e-022 |
| 4 | 76.99 | CG7379-PA | 1.3e-019 |

**NLS** (No hits)

**PHD** (5 proteins in drosophila with PHD-like domains)

| *RANK* | *SCORE* | *LOCUS* | *E_VALUE* |
| --- | --- | --- | --- |
| 1 | 119.70 | CG9293-PB | 1.8e-032 |
| 2 | 119.70 | CG9293-PA | 1.8e-032 |
| 3 | 118.35 | Ing3-PA | 4.6e-032 |
| 4 | 106.14 | CG7379-PA | 2.2e-028 |
| 5 | 84.85 | MESR4-PA | 5.6e-022 *[Weak yeast homolog YNL097C: “Chromatin remodeling protein”]* |

**PIM** (No hits)

Drosophila ING Protein Interactions

(fly ING interactors, some with yeast homologs found in Krogan *et al.* interaction set)

Interaction confidence source: Giot *et al.* unless otherwise noted

**CG7379 (ING2-like protein in drosophila)**

- CG18076 a.k.a. Shot (conf=0.98)
- CG2715 a.k.a. Syx4 (conf=0.76)
- CG5802 (conf=0.70)
- CG8609 a.k.a. Trap36, med4 (conf=0.62)
- CG8857 (conf=0.61)
- CG1478 a.k.a. Cp36 (conf=0.58)
- CG1478 a.k.a. Cp36 (conf=0.58)
- CG13164 a.k.a. Sip1, CG3552, CG4846 a.k.a. Beat-Ia, CG5068, CG7981 (conf=0.44)
- CG1554 a.k.a. RpII215, CG11006 (conf=0.35)
- CG14895 a.k.a. Pak3, CG4187, CG4383(conf=0.32)
- CG4068 (conf=0.25)
- CG7911 (conf=0.24)
- CG8983 a.k.a. Erp60 (conf=0.22)
- CG5630, CG5840 (conf=0.19)
- CG9495 a.k.a. Scm (conf=0.16)
- CG3997 a.k.a. RpL39, CG9066 (conf=0.11)
- CG4656 (conf=0.09)
- CG2746 a.k.a. RpL19 (conf=0.03)

Sip1 (CG13164) has some weak homologs in the yeast data, based on conserved coil-coil patterns:

YKR095W myosin like protein 9e-14

YDL058W ER-Golgi vesicle-tethering protein 1e-13

YLR309C grip domain 1e-11

YHR023W Myosin heavy chain 1e-10

YFL008W Structural maintenance chromosome prot. 1e-10 <- on interest list (0.012): SMC1 (human)

RpII215 (CG1554) has yeast homologs:

YOR116C rna polymerase III large subunit 0.0

YOR341W rna polymerase I large subunit 3e-9

Pak3 has several interesting homologs in the yeast data:

YNL298W Serine/threonine protein kinase 3e-75 <- not shared with human

YOL113W Serine/threonine protein kinase 2e-71 <- shared in human, fly, yeast:

p21 activated kinase 1B

YJL095W mapkkk (map kinase kinase) SSK2 4e-36 <- not shared with human

YCR073C mekk and related serine/threonine p.k.'s 1e-31 <- shared in human, fly, yeast:

MAP3K4

YDR507C Serine/threonine protein kinase 7e-29 <- on interest list (0.012): human ill-defined

YDR507C Serine/threonine protein kinase 7e-29 <- on interest list (0.012): human ill-defined

YPL150W Serine/threonine protein kinase 2e-26 <- on interest list (0.012): PAR-1A(human)

YLR113W Mitogen-activated protein kinase 3e-25 <- HOG1 on interest list (0.014): p38a (human)

“p21 activated kinase 1B” is a better homolog, but “p38a” is a better yeast

interaction score

Erp60

YCL043C “Protein disulfide isomerase” 8e-49.

**CG9293 (ING1b-like protein in fly)**

- CG32809 (conf=0.99)
- CG15658 (conf=0.82)
- CG11061 a.k.a. GM130 (conf=0.71)
- CG17244 (conf=0.33)
- CG7997 (conf=0.26)

**CG11061 weakly hits two SMCs (e-6).**

**Ing3 (source: PIMRider) (ING3-like protein in fly)**

- CG6632) – sec5 (p=0.14)
- cdc37 a.k.a. CG12019 (conf=0.48)
- hbs1 a.k.a. CG1898, CG9527 (conf=0.22)
- CG4771 (conf=0.19)
- CG18584 (sec5), CG31211 (conf=0.03)

cdc37 has weak yeast homolog

YDR168W “Cell division cycle 37 protein CDC37”, but YDR168W hits both YNG2 (0.012) and PHO23 (0.012) in the Krogan data

hbs1 has yeast homologs

YDR172W “Polypeptide release factor 3”

YPR080W “Translation elongation factor EF-1a”

sec5, CG9527 and CG4771 have no yeast homologs.

**MESR4 (CG4903)**

- TIVAMP (p=0.09)
- RAD51 (p=0.15) (source: PIMRider)

Neither RAD51 nor TIVAMP has a yeast homolog.

ING family Domain Hits in C. elegans (worm ING homologs)

No LZL, PCR, PIM or NLS hits.

**PHD (4 proteins in worm with PHD-like domains)**

| *RANK* | *SCORE* | *LOCUS* | *E_VALUE* |
| --- | --- | --- | --- |
| 1 | 90.22 | Y51H1A.4 | 1.6e-23 *[Human P33ING1B homolog (source: wormbase)]* |
| 2 | 74.89 | T06A10.4 | 6.6e-19 *[Chromatin remodeling protein (source: wormbase, KOGs)]* |
| 3 | 32.15 | C11G6.3 | 7.6e-07 *[same as previous]* |
| 4 | 29.73 | F26H11.2 | 2.0e-06 *[nurf-1 nucleosome remodeling factor (source: wormbase)]* |

C. elegans ING Protein Interactions

(worm ING interactors, some with yeast homologs found in Krogan et al. interaction set)

**Y51H1A.4 (ING1b-like protein in worm)**

- C47D12.1 (INTEROLOG, marginal, source: Li et al.)
- ZK849.2 (CORE_2, source: Li et al.)
- C47D12.1
- ZK849.2

C47D12.1 has yeast homolog

YHR099W “Phosphatidylinositol kinase”

**T06A10.4** no meaningful interactions.

**C11G6.3** no meaningful interactions.

**F26H11.2 (very weak ING-like PHD domain containing protein in worm)**

- isw-1 (predicted, conf=0.84, source: Zhong & Sternberg)

isw-1 has yeast homolog

YOR304W “Superfamily II DNA/RNA helicase”
